# Planned primary health care asthma contacts during 12-year follow-up after Finnish National Asthma Programme: focus on spirometry

**DOI:** 10.1038/s41533-020-0166-2

**Published:** 2020-03-20

**Authors:** Jaana Takala, Pinja Ilmarinen, Leena E. Tuomisto, Iida Vähätalo, Onni Niemelä, Hannu Kankaanranta

**Affiliations:** 1 Seinäjoki Health Care Centre, Seinäjoki, Finland; 2 Department of Respiratory Medicine, Seinäjoki Central Hospital, Seinäjoki, Finland; 3 Department of Laboratory Medicine, Seinäjoki Central Hospital, Seinäjoki, Finland; 4 Tampere University, Tampere, Finland; 5 Department of Respiratory Medicine, Faculty of Medicine and Health Technology, Tampere University, Tampere, Finland

**Keywords:** Asthma, Health policy, Clinical trial design, Rehabilitation, Population screening

## Abstract

Primary health care (PHC) providers are at the front line of asthma management. To evaluate how planned asthma follow-up occurred in PHC and whether lung function tests were used, 203 patients were followed for 12 years as part of a real-life asthma cohort Seinäjoki Adult Asthma Study (SAAS). A total of 152 patients had visits in PHC attending on average to four planned contacts during 12-year follow-up corresponding to one visit every third year. National guideline recommends annual visits. Patients with ≥4 contacts seemed to have more difficult asthma and better adherence to inhaled corticosteroid medication. Lung function tests were performed on average in 87.5% of annual planned follow-up contacts. Spirometry was performed in 70%, 71% and 97% of all contacts depending on whether it was a contact to GP, nurse or both. Overall, the frequency of follow-up contacts was insufficient but PHC adherence to lung function testing was excellent.

## Introduction

Asthma is a common, heterogeneous disease, causing considerable morbidity affecting all age groups^1^. Adherence to international and national guidelines in asthma seems to be highly variable^2–5^. It is logical to assume that if clinical guidelines were better adopted it would also lead to better patient outcomes.

Asthma prevalence is still increasing also in Finland^1,6^, and the most of asthma cases are diagnosed at adult age^7,8^. Remission of adult-onset asthma is rare^9,10^. There are many possible reasons for poor asthma control and high symptom burden such as allergic or chronic rhinitis, smoking, comorbidities, obesity and low initial lung function as well as problems in inhalation techniques and adherence to asthma medication^1,11,12^. Patients with both systemic inflammation and comorbidity have been shown to have the poorest outcome in asthma^13^. To improve asthma control and outcomes, it is crucial that the routine follow-up contacts in primary health care (PHC) are performed according to a high standard, and there is a need to pay attention to the quality of these contacts^2,14^.

Finland was one of the first countries to implement a national asthma programme^15^. The main goals of the Finnish Asthma Programme (1994–2004) were to improve national asthma management, prevent an increase in costs and decrease the burden of asthma to individuals and society^16,17^. One of the main objectives of the programme was to strengthen the role of PHC in the prevention, diagnosis and long-term therapy of asthma^15–19^. The Finnish Asthma Programme emphasized measures to confirm asthma diagnosis by lung function tests, to follow patients regularly and to monitor asthma control also by lung function tests intermittently^15,16^. To achieve these objectives, nurses in the PHC were trained to perform spirometry and general practitioners (GP) to interpret the result. In 2001, spirometry was available in 95% of Finnish health care centres^20^.

To our knowledge, no previous long-term follow-up studies exist on the occurrence of planned asthma follow-up contacts in PHC and use of lung function tests during the long-term follow-up of asthma. Thus the main aim of this study was to describe how planned asthma follow-up contacts occurred in PHC and to evaluate the use of objective lung function tests (spirometry and peak flow monitoring) in the long-term follow-up of asthma patients. The second aim was to evaluate the use of lung function tests depending on who encounters the patient: GP, nurse, or both.

## Results

### Characteristics of the study population

**Fig. 1:**
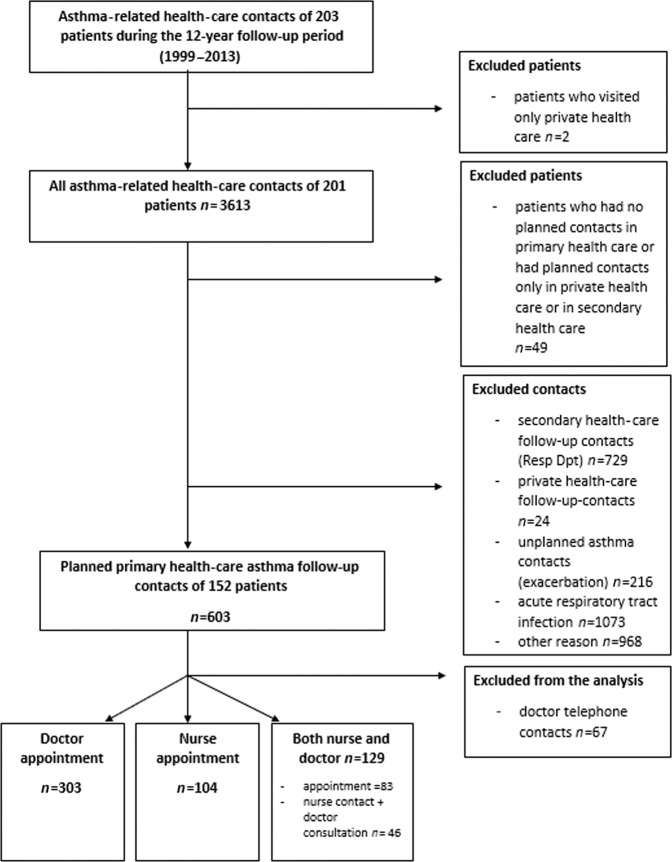
Study profile. The flowchart of the study.

The current study is a part of the real-life adult asthma cohort, Seinäjoki Adult Asthma Study (SAAS), in which 203 patients were followed for 12 years (1999–2013) after diagnosis of new-onset adult asthma^21^. The exclusion and inclusion criteria of the SAAS study are shown in eTable 1. Out of the total of 203 patients, 152 participated in planned PHC asthma follow-up contacts. Forty-nine patients were excluded because of not having planned follow-ups in PHC or having them only in private health care or in respiratory department (Fig. 1). Most of the patients with planned PHC asthma follow-up contacts were females (Table 1). At follow-up visit, mean age was 59 years and every second patient had smoking history. Approximately one third of the patients had uncontrolled asthma according to Global Initiative for Asthma (GINA) 2010^22^. The main characteristics of the study population at follow-up visit are shown in Table 1.

**Table 1:** Basic characteristics of patients having planned asthma follow-up contacts in primary care at 12-year follow-up visit.

	Patients having asthma follow-up contacts in primary care
Number of patients	152
Female, *n* (%)	96 (63.2)
Age	59 (13)
BMI	28.5 (5.9)
Smokers (ex or current), *n* (%)	76 (50.0)
Atopic, *n* (%)^a^	51 (37.2)
Rhinitis, *n* (%)	107 (71.8)
Uncontrolled asthma, *n* (%)^b^	46 (30.3)
Daily LABA in use, *n* (%)	78 (51.3)
Daily add-on drug in use, *n* (%)	85 (83.3)
Daily ICS in use, *n* (%)	125 (81.2)
Daily SABA in use, *n* (%)	21 (13.8)
≥1 oral corticosteroid course during 12-year follow-up, *n* (%)	50 (33.6)
Pre-BD FEV_1_ (%)	87 (17)
Post-BD FEV_1_ (%)	91 (17)
Pre-BD FEV_1_/FVC	0.74 (0.67–0.79)
Post-BD FEV_1_/FVC	0.76 (0.70–0.80)
FeNO (ppb)	11 (5–19)
Blood neutrophils (×10^9^/l)	3.7 (2.8–4.7)
Blood eosinophils (×10^9^/l)	0.15 (0.10–0.27)
Total IgE (kU/l)	61 (23–154)
ACO (post-FEV_1_/FVC < 0.7 and pack-years ≥10), *n* (%)	19 (12.6)
ACT score	21 (19–24)

If not otherwise mentioned, data shown are mean (SD) or median (25th–75th percentiles).

*BMI* Body Mass Index, *LABA* long-acting β2-agonist, *Add-on drug* long-acting β2-agonist, leukotriene receptor antagonist, theophylline and/or tiotropium in daily use, *ICS* inhaled corticosteroid, *SABA* short-acting β2-agonist, *BD* bronchodilator, *FEV*_*1*_ forced expiratory volume in 1 s, *FVC* forced vital capacity, *FeNO* fraction of NO in exhaled air, *ACO* asthma–COPD overlap, *ACT* asthma control test.

^a^At least one positive skin prick test of common allergens.

^b^Assessment of asthma control was performed according to the Global Initiative for Asthma (GINA) 2010 report.

### The distribution of the planned follow-up contacts in primary care

**Fig. 2:**
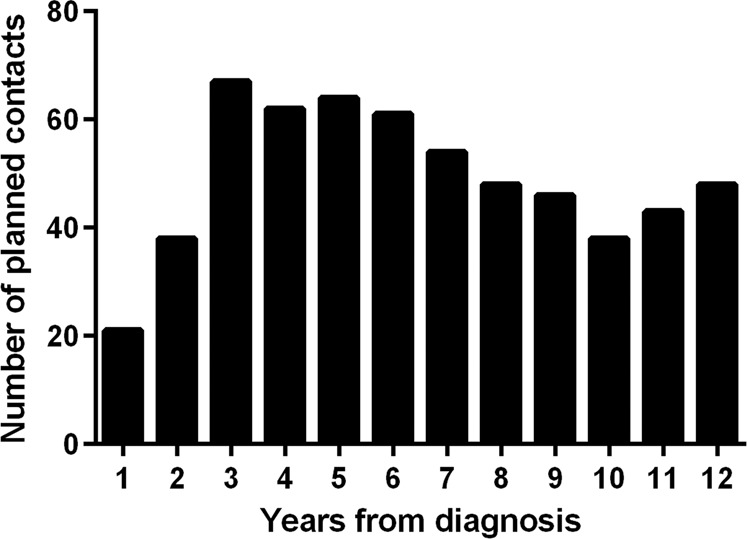
The distribution of planned contacts in primary care during 12-year follow-up. Total number of planned contacts was 603.

The number of all planned asthma follow-up contacts in PHC was 603. Thus, on average, each patient (*n* = 152) had approximately four planned contacts during the 12-year follow-up period. During the years 1–12 after diagnosis, annual number of planned contacts varied from 21 to 67 (Fig. 2). The annual average of planned contacts was 50, i.e. every third patient attended a planned visit each year.

### Differences between patients having <4 or ≥4 planned contacts

The patients participating in planned follow-ups (*n* = 152) were divided into two groups according to the number of planned asthma follow-up contacts in PHC (<4 vs. ≥4 follow-up contacts): 84 patients had <4 [median 1 (interquartile range (IQR) 1–2)] and 68 patients had at least 4 [median 6 (IQR 4–8)] planned follow-up contacts during the 12-year follow-up period. The groups with <4 vs. ≥4 follow-up visits showed no difference regarding gender, age, smoking, lung function, markers of inflammation [blood eosinophils, neutrophils, immunoglobulin E (IgE) or fraction of NO in exhaled air (FeNO)] or proportion of severe asthma according to ERS/ATS 2014^23^ (Table 2). Approximately one third of the patients in both groups had uncontrolled asthma according to GINA 2010 (Table 2)^22^. Patients with higher number of planned follow-up visits (≥4) had more often inhaled corticosteroid (ICS) medication in daily use and their adherence to ICS medication over 12 years was higher. This group had also higher number of all asthma-related health care visits and were more often in working life (Table 2). No significant differences were found in lung function or other parameters at the baseline (eTable 2).

**Table 2:** Characteristics of the study groups at 12-year follow-up visit.

	Planned PHC follow-up contacts ≥4	Planned PHC follow-up contacts <4	*P* value
Number of patients	68	84	
Female, *n* (%)	43 (63.2)	53 (63.1)	0.986
Age	59 (12.8)	60 (13.4)	0.641
BMI	27.3 (23.6–30.9)	28.1 (25.1–31.7)	0.152
Smokers (ex/current), *n* (%)	30 (44.1)	46 (54.8)	0.192
Pack-years	19 (9–32)	15 (4–28)	0.233
Rhinitis, *n* (%)	48 (72.7)	59 (71.1)	0.825
Uncontrolled asthma, *n* (%)^a^	21 (30.9)	25 (29.8)	0.510
Severe asthma, *n* (%)^b^	5 (7.4)	4 (4.8)	0.501
Daily ICS in use, *n* (%)	63 (92.6)	62 (73.8)	**0.003**
ICS dose of daily users (budesonide eq. µg)	800 (400–1000)	800 (400–1000)	1.000
ICS, *n* (%)			
At high dose	23 (39.7)	18 (25.0)	0.074
At medium dose	16 (27.6)	13 (18.1)	0.194
Total adherence in ICS medication during 12 years	82.1 (34.7)	68.1 (37.3)	**0.025**
Daily LABA in use, *n* (%)	40 (58.8)	38 (45.2)	0.096
Daily SABA in use, *n* (%)	9 (13.2)	12 (14.3)	0.852
Daily add-on drug in use, *n* (%)	43 (63.2)	42 (50.0)	0.102
≥1 oral corticosteroid course for asthma during 12-year follow-up, *n* (%)	24 (35.8)	26 (31.7)	0.597
Hospitalizations ≥1, *n* (%)	17 (25.0)	22 (26.2)	0.867
ACO (post-FEV_1_/FVC < 0.7 and pack-years ≥10), *n* (%)	7 (10.4)	12 (14.3)	0.480
ACT score	21 (19–24)	22 (20–24)	0.726
Blood eosinophils (×10^9^/l)	0.15 (0.09–0.27)	0.16 (0.10–0.29)	0.429
Blood neutrophils (×10^9^/l)	3.9 (2.7–4.7)	3.6 (2.8–4.7)	0.564
Total IgE (kU/l)	71 (26–161)	52 (22–150)	0.485
FeNO (ppb)	11 (5–19)	12 (5–19)	0.467
Pre-BD FVC (%)	97.5 (14.7)	99.6 (14.3)	0.388
Pre-BD FEV_1_ (%)	85.5 (18.0)	88.8 (16.3)	0.240
Post-BD FVC (%)	98.4 (15.0)	101.2 (14.6)	0.243
Post-BD FEV_1_ (%)	88.5 (17.9)	92.5 (15.8)	0.149
Post-BD FEV_1_/FVC	0.74 (0.69–0.80)	0.77 (0.71–0.81)	0.197
Annual change in lung function from Max_0–2.5_ to follow-up			
FEV_1_ (ml/year)	−45.6 (37.2)	−46.0 (29.1)	0.939
FEV_1_ %/year	−0.53 (1.09)	−0.44 (0.89)	0.565
Comorbidities	1.0 (0–2.0)	1.0 (0–3.0)	0.103
In working life, *n* (%)	36 (52.9)	30 (35.7)	**0.033**
Time of education ≥12 years, *n* (%)	23 (33.8)	17 (20.2)	0.059
All asthma-related health care visits during 12-year follow-up	19 (13–26)	14 (9–20)	**0.001**
Unplanned visits	3.5 (1–11)	4.0 (1–10)	0.945

If not otherwise mentioned, data shown are mean (SD) or median (25th–75th) percentiles. Statistically significant *P* values are presented in bold. Annual change in FEV_1_ or FVC from point of maximal lung function within 2.5 years after start of therapy to the 12-year follow-up visit.

*PHC* primary health care, *BMI* Body Mass Index, *ICS* inhaled corticosteroid, *LABA* long-acting β2-agonist, *SABA* short-acting β2-agonist, *Add-on drug* long-acting β2-agonist, leukotriene receptor antagonist, theophylline and/or tiotropium in daily use, *ACO* asthma–COPD overlap, *ACT* asthma control test, *FeNO* fraction of NO in exhaled air, *BD* bronchodilator, *FVC* forced vital capacity, *FEV*_*1*_ forced expiratory volume in 1 s.

^a^Assessment of asthma control was performed according to the Global Initiative for Asthma (GINA) 2010 report.

^b^Assessment of asthma severity was performed according to the ERS/ATS severe asthma guideline 2014.

### Lung function tests in planned follow-up contacts

**Fig. 3:**
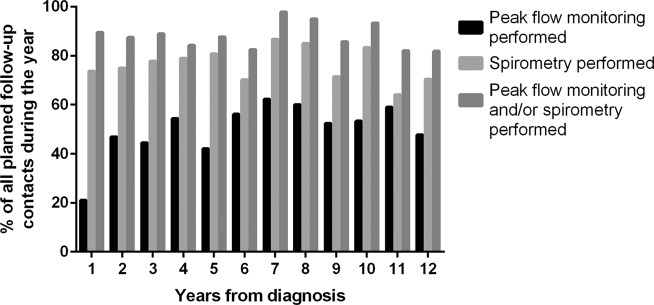
Percentage of lung function tests performed in planned follow-up contacts in primary health care. The data are presented as percentage of all annual planned contacts. Total amount of planned contacts during 12-year follow-up was 536.

To evaluate whether spirometry or peak flow monitoring were used in the follow-up of asthma as suggested by the guidelines, we collected information from the planned follow-up visits (*n* = 603). We excluded 67 follow-up contacts related to planned GP telephone contacts only. Thus, out of the total 603 contacts, we included 536 planned PHC follow-up contacts where patient encountered GP, nurse or both. Spirometry, peak flow monitoring or both were performed in 87.5% of these contacts. During the 12-year follow-up, peak flow monitoring was carried out in 51.7% of the contacts and spirometry in 76.1% of the contacts. Incomplete peak flow monitoring was excluded. The annual percentages of performed lung function tests in planned follow-up contacts (*n* = 536) are shown in Fig. 3. There was no sign of a decrease in performance of lung function testing during the 12-year follow-up.

### Lung function tests in planned follow-up contacts according to the health care professional

To evaluate whether differences exist in the use of lung function tests according to who encounters the patient in the follow-up contact, we divided the total amount of the follow-up contacts (*n* = 536) into three groups (Fig. 1). Out of all the planned follow-up contacts, 303 were GP contacts, 104 were asthma-nurse contacts and in 83 contacts patient met first nurse and GP thereafter. In 46 contacts, nurse met patient and then consulted GP, and these contacts were included to the last group (total number of combined GP and nurse contacts *n* = 129).

**Fig. 4:**
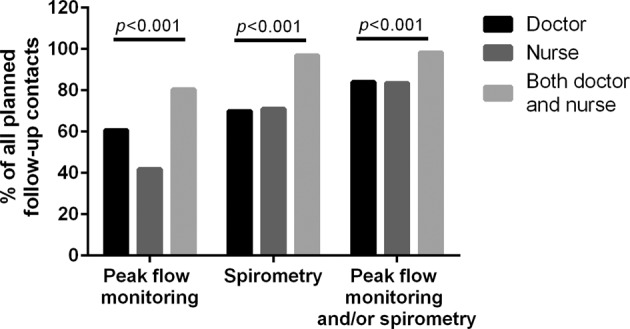
Percentage of lung function tests performed according to the health care professional encountering the patient in primary health care. Percentage of performed lung function tests in planned contacts according to professionals during the 12-year follow-up period. Number of contacts with GP was 303, 104 with nurse and 129 with both doctor and nurse.

We found that peak flow monitoring, spirometry or both were done in 98.4% of all planned asthma contacts if patient encountered both nurse and GP. Spirometry was done more often than peak flow monitoring through the whole follow-up period irrespective of who encountered the patient in the planned follow-up contact. Lung function tests were performed more often if patient met both doctor and nurse when compared to encountering either alone (Fig. 4).

## Discussion

To the best of our knowledge, no previous long-term follow-up studies exist on the occurrence of planned asthma follow-up contacts in PHC and use of lung function tests during the long-term follow-up of asthma. In this 12-year real-life follow-up study, we found that each patient had on average 4 planned asthma contacts in PHC during the follow-up period corresponding to a frequency of 1 visit every third year while the national guideline recommended annual contacts with nurse or GP. Adherence to lung function tests, especially to spirometry, as a part of assessing asthma control was excellent. Spirometry, peak flow monitoring or both were performed in 87.5% of all planned contacts, spirometry in 76.1% and peak flow monitoring in 51.7% of contacts. If both professionals were involved in follow-up visit, lung function tests were done in almost every planned asthma contact. These results suggest that in Finland the frequency of asthma follow-up contacts is insufficient but the PHC adherence to lung function test performance is at high level.

According to Finnish guidelines^16,24^ and current GINA report, asthma patient should have regular review by health care provider^1^. In many studies, it has been suggested that adherence to recommended regular follow-up is insufficient^5,17,25^, and many patients are lost to follow-up^26^. In these studies, conclusions have mostly been made based on relatively short follow-up or based on asthma-related visits or planned contacts in PHC during the previous year. The recommendation of the Finnish Asthma Programme^15,16^ was that patients should continue visits with health care professionals yearly even if asthma is controlled. We found that the given recommendation on asthma follow-up contact frequency was not followed even if patients were informed about the importance to continue long-term visits in PHC. In our study, 49 out of 203 patients were excluded because of not having planned follow-ups in PHC and 29 of these patients did not have any follow-up contacts during the 12-year follow-up period. Total of 152 patients participated in planned asthma contacts mostly in PHC but it is possible that some of the patients had also additional asthma contacts in respiratory department or in private health care. The first two follow-up contacts after asthma diagnosis were mainly done in the respiratory department explaining why there were fewer planned contacts in PHC during the first 2 years of the follow-up period. After the first 2 years, the number of planned asthma contacts increased and slightly decreased in the middle of the follow-up period until new increase towards the end of the 12-year follow-up. The patients who had planned follow-up contacts in PHC had on average four contacts, but when using this amount as a threshold value we found that most patients (*n* = 84) had less than four planned contacts during the 12-year follow-up period showing that most of the asthma patients are not regularly visiting a doctor or nurse. Our finding is supported by the previous Swedish observational cohort study^25^ where on average every third patient visited primary care doctor because of asthma irrespective of disease severity. Previous Finnish cross-sectional study showed that in 2010 69% of asthma patients reported a scheduled visit to a physician compared to 73% in 2001^17^. Scheduled appointments to nurse reduced similarly from 28% in 2001 to 23% in 2010 while health care services had essentially remained the same^17^. However, study consisted of patients visiting pharmacies^17^, indicating that more therapy and planned follow-up adherent patients might have been selected. Recent American study showed that across all age groups 22.2% of the patients had no asthma-related visits to the primary care in the previous year. The visits that were actualized due to asthma were made for evaluation of acute symptoms, but planned asthma care visits were not found^5^. The previous and our results suggest that nonadherence to follow-up is a worldwide phenomenon. Because remission of adult-onset asthma is rare unlike in childhood asthma^9,10,27,28^, missing regular follow-up cannot be assumed to be harmless. It can be claimed that even four planned contacts during a 12-year follow-up period is too few.

Planned asthma management with systematic approach in general practice has been shown to improve asthma control^29^. We were not able to find studies considering occurrence of long-term planned follow-up in PHC and how planned contacts affect asthma control in long-term period. It is logical to assume that patients with more planned asthma contacts are having better asthma control and that regular long-term follow-up improves outcome. In our study, one third of the patients in both groups (<4 or ≥4 planned contacts) had uncontrolled asthma according to GINA 2010^22^, and there was no difference in the proportion of severe asthma according to ERS/ATS 2014^23^ and no differences in asthma control according to asthma control test (ACT) scores or lung function. These findings suggest that the frequency of asthma contacts had no effect on the level of asthma control. The group with four or more planned contacts had also more other asthma-related health care contacts. This result combined with tendency to increased use of high ICS dose and add-on drugs suggests that these patients had more persistent and difficult disease. They also had better adherence to ICS medication. Thus our results suggest that patients with more difficult asthma are more likely to participate regularly in planned follow-up contacts and they also have better adherence to medication. One could also speculate that with more regular follow-up it is possible to treat more persistent asthma to the same level with milder ones because the two contact groups did not have any significant differences in lung function, markers of inflammation or asthma control at the end of the follow-up. To support this, a Danish study showed that systematic approach in planned follow-up contacts increased the level of well-controlled asthma by 20% and reduced uncontrolled asthma by 14%^29^. Advantages of more frequent contacts were also reported recently with type 2 diabetes patients who had stopped to attend to follow-up in diabetes clinics as prescribed: with more frequent contacts, they succeeded to improve their glycaemic control in primary diabetes health care^30^. Previous results support the assumption that with regular follow-up it is possible to improve control of a persistent disease. However, we were not able to assess whether medically correct actions were taken in planned asthma follow-up contacts or whether the good adherence to ICS medication was due to more regular follow-up or more difficult asthma or both of them. Previous study of the SAAS cohort showed that cumulative dose of ICS increased during the 12-year follow-up period and prescription discontinuation was rare^31^. Good adherence to asthma therapy has been suggested to improve the clinical outcomes and to lessen health care costs^32^.

Access to asthma follow-up visits has shown significant regional variation in Finland depending for example on the municipal service system and resources^33^. In our study, patients with four or more planned contacts were more often in working life even though the mean age of the two groups was similar. One explanation may be that employees may have had better access to PHC services in Finland, as previously suggested^34,35^, because of the ability to use both occupational and PHC services. There are probably many patient-related issues affecting adherence to asthma follow-up including attitudes, personal resources, ability and asthma symptoms. Many patients with asthma do not regard themselves as sick and are not concerned about their condition^36^, and it could also be one reason to miss follow-up in our study. In previous studies, patients lost to follow-up have been younger and have had clinical features of less severe asthma at the time of diagnosis, with similar findings also in studies concerning adherence to asthma medications^26,32,37^. In our study, age, sex or lung function at baseline was not associated with less frequent follow-up. In the group of less than four planned contacts, almost 74% of the patients reported daily ICS use but the median daily ICS dose was 800 µg indicating that most of the patients using ICS were treated with moderate-to-high doses.

Guidelines recommend that assessment of asthma should include evaluation of symptom control, future risk of adverse outcomes, treatment issues such as inhaler technique and adherence, side effects, smoking and comorbidities^1,24^. There is no universal common consensus about all aspects and contents of asthma control visits for example for lung function testing. Current GINA report recommends objective lung function measurements as necessary for initial diagnosis of asthma as well as long-term monitoring of asthma^1^. Previous studies have shown that reliance on patient-reported clinical symptoms^38–41^ or ACT score can lead to overestimation of asthma control^41,42^. Inclusion of spirometry in the assessment guarantees more accurate monitoring of asthma control^38–41^ without input from secondary care^43^. Objective lung function measurements are not comprehensively used in asthma diagnostics^44^ and monitoring^2,3,45^, despite several studies^38–41^ and guidelines^1^ supporting their use. A Swedish study showed that one third of the patients with asthma visiting PHC during initial visits and approximately half of the patients during follow-up visits had a clinical evaluation, including spirometry or peak flow monitoring, in agreement with recommendations^2^. In Germany, 57% of physicians used spirometry as a part of assessing asthma control when proportion was 46% in France, 47% in Australia, 28% in Canada, 54% in China and 24% in Japan^3^. In contrast, our results show that in Finland spirometry was performed in >76% of all scheduled contacts.

To the best of our knowledge, there are no previous studies investigating the longitudinal lung function follow-up of adult asthma patients in PHC. We found that spirometry, peak flow monitoring or both were performed in almost 88% of all planned follow-up contacts. When both professionals took part in the visit, lung function tests were carried out in almost every planned contact. Utilization of spirometry was higher compared with peak flow monitoring during the entire follow-up. In Finland due to the law of special reimbursement for chronic asthma medication, it has been crucial for decades to confirm asthma diagnosis by objective lung function tests, but continuous follow-up of lung function tests has not been required for the reimbursement. In the PHC, the use of spirometry increased significantly after introduction of both the national programmes of asthma (1994–2004) and chronic obstructive pulmonary disease (COPD; 1997–2007) and the current asthma care guideline (2000–)^46^. To enhance the implementation of the asthma programme, regional guidance was also available in 79% of the Finnish health care centres in 2001^20,47^. The quality of the Finnish PHC spirometry curves has been found good in 78–80% of cases^48^. As shown also in the previous study of pre-diagnostic lung function tests in the same area^49^, the current study of the post-diagnostic use of lung function tests support adherence to the national and regional asthma guidelines.

The Current Finnish Guideline recommends that asthma patient should have an annual planned contact with nurse or GP if asthma control is otherwise good and that the appointment with GP should be at least every third or fifth year^24^. Based on the evaluation of the results of the Finnish Asthma Programme, it was recommended that the role of asthma nurses should be further strengthened so that educated nurses could perform most of the annual asthma follow-up contacts^16^. Our study showed that this was not reached while only approximately 17% (*n* = 104) of all planned contacts were nurses’ and most of the patients had overall less than four planned contacts during the follow-up period. Similarly, in previous studies most of the planned visits of asthma patients were doctor appointments^17,25^. According to a previous Finnish study, respiratory nurses in PHC tend to lack appropriate time in relation to number of respiratory patients when they also take care of other patients and tasks^33^. In our study, spirometry, peak flow monitoring or both was performed in almost every planned contact if patient encountered both nurse and GP. This suggests that planned asthma follow-up contact may benefit from the involvement of both professionals^50^. In a Danish study^29^, planned asthma management by both nurse and doctor participating with systematic approach improved asthma control. In a previous review, nurse-led care did not have any differences when compared to physician-led management of asthma^51^, but because the review included only one study with uncontrolled patients and was based on relatively small number of studies that the results cannot be directly applied to primary care practice where patients are often multimorbid and have often uncontrolled disease.

Our study has several strengths. The diagnosis of asthma was made by a respiratory physician and the diagnosis was based on typical symptoms and objective lung function measurements showing reversibility of airway obstruction. Smokers and patients with comorbidities were not excluded. Therefore, this study population well represents a typical PHC population with asthma^21^. Possible weakness of our study is that our results may not represent entire Finland. There may be regional imbalance for example in the frequency of spirometry or planned follow-up contacts. We were not able to assess what kind of conclusions were made based on the lung function tests and how these conclusions affected on therapy and asthma control. Also, skills of GPs to interpret spirometry were not estimated. We were not able to assess how often spirometry revealed a clinical issue that was not emerged by measuring asthma control with ACT because in Finland ACT was gradually introduced around 2010.

Evidence-based medicine and guidelines have improved the quality of health care, but still suboptimal adherence to care guidelines is a common worldwide problem seen not only with asthma^2–5^ and chronic obstructive pulmonary disease^52–54^ but also with other common chronic conditions, such as cardiovascular diseases and diabetes^55–59^. GPs generally deal with multimorbid patients. It could be argued that asthma may lack appropriate attention and follow-up with patients with multimorbidity, as recently found with COPD^54^. Based on our results, it is essential to pay more attention to asthma follow-up not only when the frequency of planned contacts is insufficient but also when many patients choose not to participate in follow-up. In the Finnish health care system, arranging the follow-up contact is primarily the patients’ responsibility as most often no recall systems are used in PHC. It is essential to pay more attention to occurrence of planned follow-up contacts during the routine prescribing or dispensing. Adequate resources, including respiratory nurses, in PHC should be guaranteed because it has influence both on management of regular follow-up of asthma and other chronic conditions and on availability of health care services. The role of respiratory nurses should be strengthened so that they could focus more on respiratory patients and their follow-up. It can be argued whether every patient needs an annual asthma follow-up contact if asthma is mild and otherwise in control. In the future, identification of asthma phenotype may enable to determine the optimal follow-up frequency for different patients^12^. More research is needed to evaluate how other essential factors such as smoking and comorbidities associated with asthma control are managed in follow-up contacts in long-term period.

In conclusion, we showed that PHC adherence to lung function measurements, especially to spirometry, as a part of assessing asthma control is good in Finland. The frequency of asthma follow-up contacts in PHC is insufficient when only every third patient was attending a planned visit each year. We showed that adherence to therapy may be better if patients have more planned contacts. In the future, it is necessary to pay more attention to asthma follow-up and characterize the population who is at a risk to drop out of asthma follow-up.

## Methods

### Study design, inclusion and exclusion criteria

The present study was a part of SAAS, which is a single-centre (Department of Respiratory Medicine, Seinäjoki Central Hospital, Seinäjoki, Finland) 12-year real-life follow-up study of patients with new-onset asthma diagnosed at adult age (≥15 years). The details of the SAAS study protocol with specific diagnostic criteria has been published separately previously^21^. This study is registered at www.ClinicalTrials.gov with identifier number NCT02733016.

In the original study, cohort patients (*n* = 257) were recruited between October 1999 and April 2002 from the diagnostic visit in Seinäjoki Central Hospital respiratory department. Diagnosis of new-onset asthma was made by a respiratory physician based on typical symptoms and was confirmed by objective lung function measurements^9,12,21^. Smokers and patients with concomitant COPD or other comorbidities were not excluded (Supplementary Table 1). After the diagnosis was confirmed and the medication started, the patients were treated and monitored by their personal physicians mostly in PHC according to the Finnish National Asthma Programme^15,16^.

After 12 years (mean 12.2, range 10.8–13.9), a total of 203 patients completed a follow-up visit in respiratory department. Asthma status, disease control, comorbidities and medication were evaluated using structured questionnaires (Airways Questionnaire 20 (AQ20) and ATC), and lung function was measured. The participants of the follow-up visit gave written informed consent to the study protocol approved by the Ethics committee of Tampere University Hospital, Tampere, Finland (R12122). In addition to the data gathered at these visits, all data of asthma-related health care contacts during 12-year period was collected from PHC, occupational health care, private clinics and hospitals as previously prescribed^9,12,21^. The flowchart of the study is shown in Supplementary Fig. 1.

In the present study, all asthma-related health care contacts of the 203 patients during the 12-year follow-up period were explored. Two of the patients were excluded in the beginning because they visited only in private health care. The rest 201 patients had 3616 asthma-related health care contacts. Of those, we included planned PHC (public health care centres and occupational health care) asthma follow-up contacts of 152 patients, the total number of contacts being 603 (Fig. 1). Out of the rest 49 patients, 20 arranged their follow-up in private health care and 29 patients did not have any planned follow-up between the diagnostic visit and the year 2013 follow-up visit in the respiratory department. The data of 152 patients and the data gathered from their planned asthma contacts in PHC were evaluated. During the SAAS study period, all health care centres in our region had respiratory nurses and coordinator–GP responsible for the asthma management in the health care centre, yet every GP managed their own asthma patients.

### Lung function, computation of adherence, inflammatory parameters and other clinical measurements

Lung function measurements were performed with a spirometer according to international recommendations^60^. Only complete 2-week peak flow monitoring was included when evaluating the use of lung function tests. Prescribed medications and dose calculations were carried out based on the data obtained from planned asthma contacts and the dispensed ICS doses were obtained from the Finnish Social Insurance Institution that records all purchased medication from any Finnish pharmacy. Adherence to ICS medication was evaluated by comparing the patient’s dispensed doses to the prescribed doses for the whole 12-year period. Shortly, we converted all prescribed and dispensed ICS doses to budesonide equivalents and based on that information calculated annual and total 12-year adherence for each patient^61^. FeNO was measured with a portable rapid-response chemiluminescent analyser according to American Thoracic Society standards^62^ (flow rate 50 mL/s; NIOX System, Aerocrine, Solna, Sweden). Venous blood was collected, and white blood cell differential counts were determined. Total IgE levels were measured by using ImmunoCAP (Thermo Scientific, Uppsala, Sweden). Laboratory assays were performed in an accredited laboratory (SFS-EN ISO/IEC 17025:2005 and ISO 15189:2007) of Seinäjoki Central Hospital. Patients completed AQ20^63^ and ACT. Assessment of asthma control was performed according to the GINA 2010 report^22^.

### Definition of PHC

In Finland health care services are divided into PHC and specialized medical care. The country is divided into 21 hospital districts, which provide specialist medical care for the population in their area. Finland has approximately 160 health care centres and many of these consist of several branches, especially in cities. In addition, employers have an obligation to provide occupational health care for their employees. The primary aim of occupational health care is to maintain and improve work ability^64^. For example, an adult working person who has a new-onset asthma diagnosed at specialized medical care may have the ability to use either PHC services or occupational health care. In this study, we considered both planned follow-up contacts in health care centres and in occupational health care as the PHC follow-up contacts.

### Statistical analysis

Continuous data are expressed as mean (SD) for variables with normal distribution and, if skewed distribution, shown as median and 25th–75th percentiles. Shapiro–Wilk test was used to assess normality. Two-group comparisons were performed by using Student’s *t* test for continuous variables with normal distribution, Mann–Whitney test for continuous variables with skewed distribution or Pearson Chi-square test for categorized variables. Statistical analyses were performed using the SPSS software, version 25 (IBM SPSS, Armonk, NY). A *P* value < 0.05 was regarded as statistically significant. Two-sided *P* values were used.

### Reporting summary

Further information on research design is available in the Nature Research Reporting Summary linked to this article.

## Supplementary information

Supplementary Information

Reporting Summary

## Data Availability

All data generated or analysed during this study are included in this published article (and its Supplementary Information File). According to ethical permission and patient data protection laws of Finland, single patient data cannot be made available.
