# Supplementary material for: Planned primary health care asthma contacts during 12-year follow-up after Finnish National Asthma Programme: focus on spirometry
Source: NPJ Prim Care Respir Med. 2020 Mar 20;30:8. doi: 10.1038/s41533-020-0166-2 (PMC7083866; doi:10.1038/s41533-020-0166-2)
Supplement: Supplementary file 1 — Supplementary Information [file 41533_2020_166_MOESM1_ESM.pdf]

Supplementary information

Planned primary health care asthma contacts during 12-year follow-up after Finnish National Asthma Programme: focus on spirometry

Jaana Takala, MD, GP[1,2*], Pinja Ilmarinen, PhD[2], Leena E. Tuomisto, MD, PhD[2], Iida Vähätalo, MSc (Pharm)[2], Onni Niemelä, MD, PhD[3,4], Hannu Kankaanranta, MD, PhD[2,5]

[1]Seinäjoki Health Care Centre, Seinäjoki, Finland

[2]Department of Respiratory Medicine, Seinäjoki Central Hospital, Seinäjoki, Finland.

[3]Department of Laboratory Medicine, Seinäjoki Central Hospital, Seinäjoki, Finland

[4]Tampere University, Tampere, Finland

[5]Department of Respiratory Medicine, Faculty of Medicine and Health Technology, Tampere University, Tampere, Finland.

Corresponding author: Dr Jaana Takala, MD, GP

Seinäjoki Health Care Centre

Koskenalantie 18

FIN-60220 Seinäjoki, FINLAND

e-mail: jaana.takala@seinajoki.fi

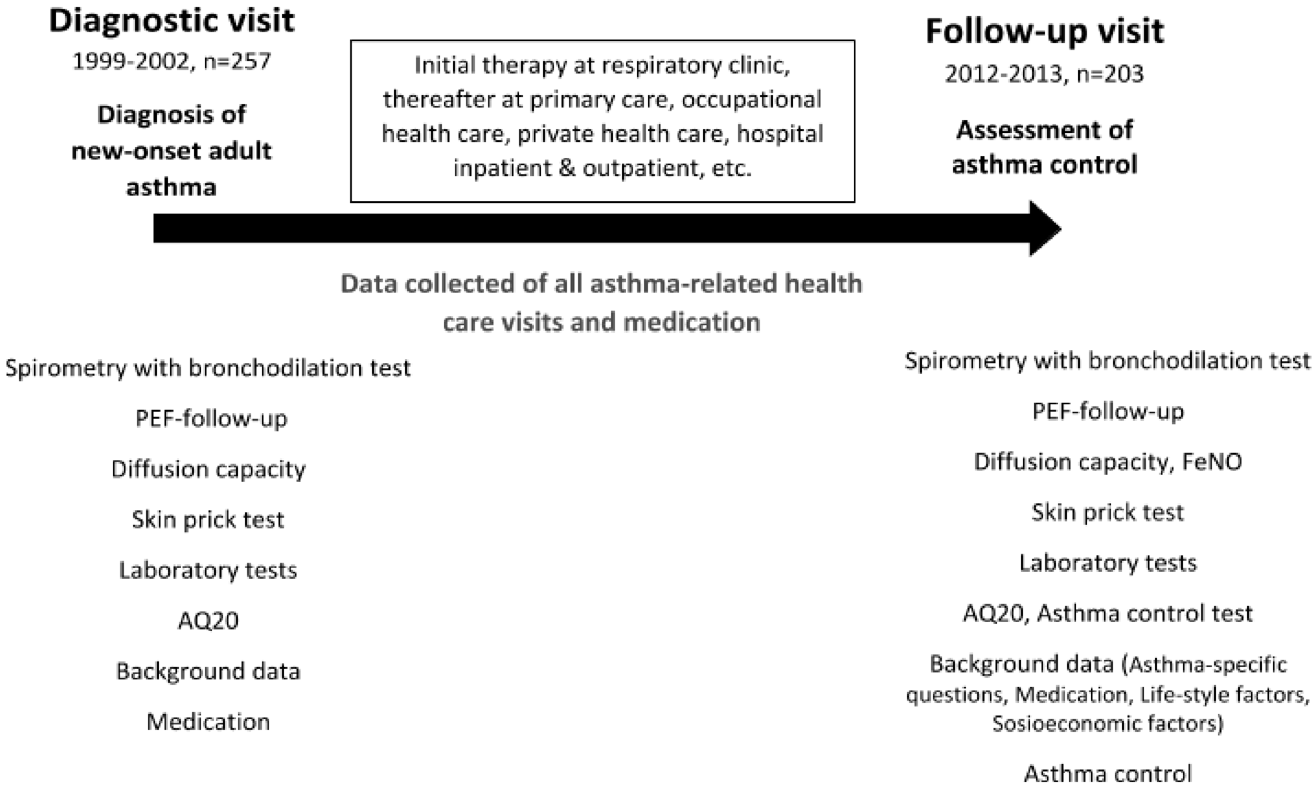

Supplementary Figure 1. Flowchart of Seinäjoki Adult Asthma Study.

Supplementary Table 1. Exclusion and inclusion criteria used in SAAS-study.

Inclusion criteria

- A diagnosis of new-onset asthma made by a respiratory specialist
- Diagnosis confirmed by at least one of the following objective lung function measurements
    - FEV_1 reversibility in spirometry of at least 15 % and 200 ml
    - Diurnal variability (⩾20%) or repeated reversibility (⩾15%/60 l/min) in PEF follow-up
    - A significant decrease in FEV_1 (15%) or PEF (20%) in response to exercise or allergen
    - A significant reversibility in FEV_1 (at least 15% and 200 ml) or significant mean PEF change in response to a trial with oral or inhaled glucocorticoids
- Symptoms of asthma
- Age ≥ 15 years

Exclusion criteria

- Physical or mental inability to provide signed informed consent
- Diagnosis of asthma below the age of 15 years

Reference: Kankaanranta H, Ilmarinen P, Kankaanranta T, et al. Seinäjoki Adult Asthma Study (SAAS): a protocol for a 12-year real-life follow-up study of new-onset asthma diagnosed at adult age and treated in primary and specialised care. *NPJ Prim. Care Respir. Med.* 2015;25:15042.

Supplementary Table 2. The baseline characteristics in the contact groups.

At study baseline	Planned primary health care follow-up contacts ≥4	Planned primary health care follow-up contacts <4	P-value
Number of patients	68	84	
BMI	26.5 (23.3-29.1)	27.2 (25.0-29.7)	0.159
Smokers (ex/current) n(%)	29 (42.6)	44 (52.4)	0.232
ACO(Post FEV_1/FVC <0.70 and pack-y≥10) n(%)	6 (8.8)	5 (6.1)	0.524
Atopic n(%)	23 (37.7)	28 (36.8)	0.917
AQ20 score	6 (3-10)	7 (4-10)	0.351
Blood eosinophils ($x10^9$/l)	0.25 (0.15-0.45)	0.30 (0.15-0.42)	0.895
Total IgE (kU/l)	90 (33-218)	73 (36-161)	0.698
Pre-BD FVC (%)	90.0 (14.1)	90.4 (16.7)	0.864
Pre-BD FEV_1 (%)	80.5 (15.6)	82.1 (18.4)	0.569
Pre-BD FEV_1/FVC	0.74 (0.08)	0.75 (0.11)	0.425
Post-BD FVC (%)	93.6 (14.0)	92.6 (15.8)	0.711
Post-BD FEV_1 (%)	87.0 (16.9)	87.8 (17.9)	0.766
Post-BD FEV_1/FVC	0.77(0.09)	0.79 (0.11)	0.316
FEV_1 Reversibility (ml)	170 (90-300)	160 (70-345)	0.859
FEV_1 Reversibility (%)	6.7 (3.3-11.5)	5.7(2.1-12.0)	0.695

If not otherwise mentioned shown are mean (SD) or median (25th -75th percentiles). BMI = Body Mass Index, ACO = asthma-COPD overlap, AQ20 = Airway Questionnaire 20, BD = bronchodilator, FEV_1 = forced expiratory volume in 1 s, FVC = forced vital capacity.
